# Effects of Ultrasonic Bending Vibration Introduced by an L-Shaped Ultrasonic Rod on the Microstructure and Properties of a 1060 Aluminum Alloy Strip Formed by Twin-Roll Casting

**DOI:** 10.3390/ma13092013

**Published:** 2020-04-25

**Authors:** Chen Shi, Gaofeng Fan, Xuqiang Mao, Daheng Mao

**Affiliations:** 1 College of Mechanical and Electrical Engineering, Central South University, Changsha 410083, China; fangaofeng@csu.edu.cn; 2 State Key Laboratory of High Performance Complex Manufacturing, Central South University, Changsha 410083, China; 3 Light Alloy Research Institute, Central South University, Changsha 410083, China; maoxuqiang@csu.edu.cn (X.M.); mdh@csu.edu.cn (D.M.)

**Keywords:** ultrasonic bending vibration, 1060 aluminum alloy, twin-roll casting, microstructure, mechanical properties

## Abstract

In order to achieve the industrial application of ultrasonic energy in the continuous casting and rolling production of aluminum alloy, a new type of L-shaped ultrasonic rod was used to introduce an ultrasonic bending vibration into the aluminum melt in the launder during the horizontal twin-roll continuous casting and rolling process of a 1060 aluminum alloy. The effects of the ultrasonic bending vibration on the microstructure and properties of the 1060 aluminum alloy cast rolling strip and its subsequent cold rolling strip were studied experimentally, and the effect of the ultrasonic-assisted refining with different amounts of Al-Ti-B refiner was explored. The results show that under the same addition amount of Al-Ti-B refiner, the ultrasonic bending vibration can refine the grains of the cast rolling strip, make the distribution of precipitates more uniform, reduce the slag inclusion defects, and improve the mechanical properties to a certain extent. The microstructure and properties of the ultrasonic cast rolling strip with 0.18 wt% Al-Ti-B refiner or 0.12 wt% Al-Ti-B refiner are better than those of the conventional cast rolling strip, but the microstructure and properties of the ultrasonic cast rolling strip with 0.09 wt% Al-Ti-B refiner are slightly worse than those of the conventional cast rolling strip. Moreover, after cold rolling, the effect of the ultrasonic bending vibration on the improvement of the microstructure and properties of the aluminum alloy strip is inherited. A comprehensive analysis shows that the use of ultrasonic energy in this paper cannot completely replace the effect of the Al-Ti-B refiner, but it can reduce the addition amount of the Al-Ti-B refiner by 1/3.

## 1. Introduction

Aluminum alloy is the most widely used non-ferrous metal structure material in the metallurgy, chemical, construction, transportation, aerospace, and weapons industries [1,2]. With the application of aluminum alloys in the high-tech field, stricter requirements are imposed on the structure and properties of aluminum alloys [3,4]. At present, grain refinement by adding an Al-Ti-B refiner is an effective way to improve the performance of aluminum alloy strips in casting and rolling production [5,6,7]. A large number of researchers have studied and improved the Al-Ti-B refiner to improve its refining efficiency [8,9,10,11]. However, the addition of the Al-Ti-B refiner will generate several undesirable by-products, including the formation of particle aggregates, local defects, and impurities; in addition, the refiner when used as a consumable also increases the cost of the casting and rolling production.

In recent years, the use of additional physical fields such as ultrasonic vibration or pulsed magneto-oscillation (PMO) to replace the refiner used to refine the grains has aroused interest among researchers [12,13,14,15,16]. Xia et al. [17] used ultrasonic vibration to treat a 3003 aluminum alloy in continuous casting and rolling and found that the effect of ultrasonic treatment was better than that of adding Al-Ti-B refiner and that the refiner could be completely replaced, reducing the production cost and improving the material properties of the strip. Shi et al. [18] applied ultrasonic vibration to the process of 8011 aluminum alloy twin roll casting and rolling, and the research results showed that through the effect of ultrasonic vibration, the grains of the strip were made smaller and the mechanical properties were improved to a certain extent. Xu et al. [19] reduced the amount of Al-Ti-B by applying a electromagnetic field to the cast rolling of the 1100 aluminum alloy. It was found that the refining effect of adding 0.1 wt% Al-Ti-B refiner under the electromagnetic field roll-casting conditions was better than that of adding 0.4 wt% Al-Ti-B refiner under the conventional roll-casting conditions.

The key to the realization of ultrasonic-assisted metal solidification forming is the introduction of the ultrasonic wave. High-energy ultrasound can cause a cavitation effect and acoustic flow effect in the melt. With the increase in ultrasonic power, the effects will be significantly enhanced [20]. Due to these effects, the homogeneity of solute elements and fluidity of the melt can be enhanced, as well as improving the grain refinement and melt degassing efficiency [21,22,23,24]. At present, most researchers use a method of directly introducing ultrasonic waves in the upper part of the melt to treat the metal melt. The ultrasonic transducer is subjected to direct thermal radiation and a thermal shock from the molten metal, which often causes the transducer to be detuned or even damaged during the casting process. In addition, the titanium alloy ultrasonic radiator in contact with the melt is easily eroded [25,26,27]. These have made it difficult to achieve long-term ultrasonic effects in metal melts.

In recent years, our research group has developed a new type of L-shaped ultrasonic rod which uses a nano ceramic radiator to avoid the ultrasonic transducer being directly affected by high-temperature heat radiation and resist the erosion of metal melt, which can meet the requirements of long-term continuous work and is conducive to industrial application. Shi et al. [28] applied the L-shaped ultrasonic device to the solidification process of a large 2A14 aluminum alloy ingot (φ830 mm × 6000 mm). The study showed that it can significantly refine the grains of the large ingot, effectively decrease the degree of solute segregation, and improve its mechanical properties. The mechanical vibration generated by the ultrasonic transducer was conducted by an L-shaped ultrasonic rod, which formed an ultrasonic bending vibration at the head of the ceramic tool and led to metal melt. Based on the industrial test, this paper studies the influence of ultrasonic bending vibrations on the microstructure and properties of a 1060 aluminum alloy strip in the casting and rolling process and explores the ultrasonic-assisted refining effect of Al-Ti-B refiner in different usage conditions.

## 2. Material and Methods

### 2.1. Experimental Equipment

**Figure 1:**
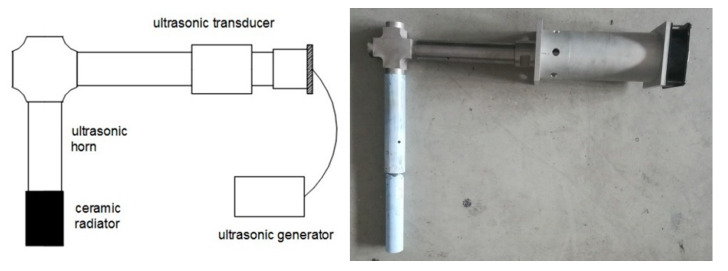
L-Shaped ultrasonic rod device.

A new self-made L-shaped ultrasonic rod was used in the experiment, as shown in Figure 1. The L-shaped ultrasonic rod was composed of a transducer, a first-level horn that transmitted longitudinal mechanical vibrations in the horizontal direction, a second-level horn that transmitted bending vibrations in the vertical direction, and a ceramic radiator. The material used for the horn was a TC4 titanium alloy, and the material used for the ceramic radiator in direct contact with the metal melt was nano-silicon nitride ceramic. The working frequency of the ultrasonic wave was 21 ± 0.2 kHz and the power range was 0–1000 W.

### 2.2. Experimental Material

The experimental material was the 1060 aluminum alloy with the chemical composition listed in Table 1.

### 2.3. Experimental Procedures

**Figure 2:**
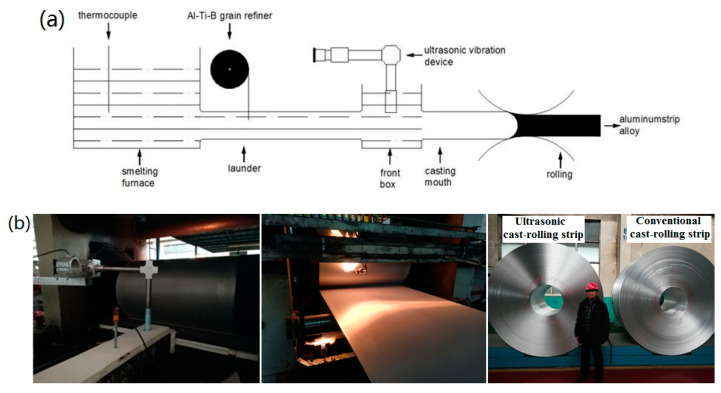
Schematic (**a**) and field picture (**b**) of the continuous casting and rolling of the 1060 aluminum alloy.

The experiment was performed on a horizontal two-roll continuous cast rolling unit. Four groups of continuous casting and rolling experiments were carried out on the 1060 aluminum alloy: (1) adding 0.18 wt% Al-Ti-B refiner, conventional casting and rolling; (2) adding 0.18 wt% Al-Ti-B refiner and applying an ultrasonic bending vibration; (3) adding 0.12 wt% Al-Ti-B refiner and applying an ultrasonic bending vibration; (4) adding 0.09 wt% Al-Ti-B refiner and applying an ultrasonic bending vibration. The process of the 1060 aluminum alloy ultrasonic-assisted twin-roll continuous casting and rolling is shown in Figure 2.

The technological parameters of the twin-roll casting unit were as follows: the roll gap was 5.7 mm, the twin roll casting speed was 0.85 m/min, the pouring temperature of the aluminum melt measured by the thermocouple was 695 °C, the temperature of the cooling water inlet in the roll was 34 °C, the water outlet was 41 °C, and the water pressure was 0.9 MPa. The parameters of the ultrasonic equipment were as follows: the power of ultrasonic wave was 800 W, the frequency was 21 ± 0.2 kHz, and the ceramic radiator was inserted into the aluminum melt surface in the launder at about 50 mm.

### 2.4. Test Method

#### 2.4.1. Microstructure Analysis of the Cast Rolling Strip Samples

The 20 × 20 × 6 mm samples were cut at a distance of 20 mm from the edge of the cast rolling strips. The samples were ground and polished in the MP-2B polishing machine and the morphology, size, and distribution of the precipitated phases were observed using a Phenom fully automatic scanning electron microscopy (Phenom-world BV, The Netherlands). Then, the electrolyte (19 mL water, 38 mL concentrated sulfuric acid, 43 mL phosphoric acid) was prepared for anodic coating. The current was controlled at 0.2 A, the temperature was controlled at 20 °C~30 °C, and the surface was observed every 30 s. After the grains appeared, the pictures of the grains on different sides of the cast rolling strip samples were obtained by a metallographic microscope (OLYMPUS DSX500 metallurgical microscope (Olympus, Japan)).

#### 2.4.2. Microstructure Analysis of the Cold Rolling Strip Samples

In order to investigate the evolution of the microstructure and properties of the cast rolling strip after cold rolling, 4 groups of cold rolling strips with 0.3 mm thickness were obtained after the cold rolling of 4 groups of cast rolling strips. The 10 mm × 20 mm rectangular samples were cut in the middle of the cold rolling strips. After the electrolytic (90 mL of alcohol, 5 mL of water, 7 mL of perchloric acid) was prepared for electrolytic polishing, the morphology, size, and distribution of the precipitated phase were observed using a Phenom fully automatic scanning electron microscope (Phenom-world BV, The Netherlands). Then, an electrolytic (40 mL of water, 1 mL of HF) was prepared for anode coating, and grain pictures were obtained through a metallographic microscope (OLYMPUS DSX500 metallurgical microscope (Olympus, Japan)).

#### 2.4.3. Test Mechanical Properties

Refer to the ASTME8M-04 metal material tensile test method for the tensile property test. Standard tensile samples were cut along the 0° direction of the strip (casting and rolling direction, RD), 45°, and 90° (transverse casting and rolling direction, TD). The tensile properties of the samples were tested at room temperature with the MTS 810 universal material testing machine (USA). The strain rate was 2 mm/min.

## 3. Results and Discussions

### 3.1. Effect of the Ultrasonic Bending Vibration on the Grain Microstructure of the 1060 Aluminum Alloy Cast Rolling Strip and Subsequent Cold Rolling Strip

**Figure 3:**
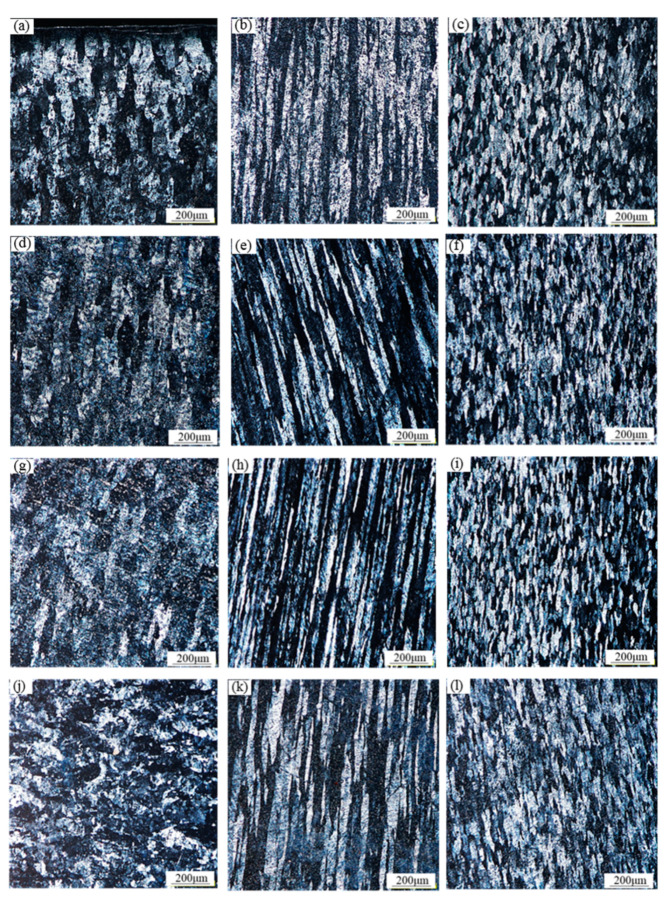
Grain microstructure of the 1060 aluminum alloy cast rolling strips: (**a**–**c**) the top surface, longitudinal section, cross section of the strip with added 0.18 wt% Al-Ti-B refiner and applied conventional treatment; (**d**–**f**) the top surface, longitudinal section, cross section of the strip with added 0.18 wt% Al-Ti-B refiner and applied ultrasonic treatment; (**g**–**i**) the top surface, longitudinal section, cross section of the strip with added 0.12 wt% Al-Ti-B refiner and applied ultrasonic treatment; (**j**–**l**) the top surface, longitudinal section, cross section of the strip with added 0.09 wt% Al-Ti-B refiner and applied ultrasonic treatment.

The microstructure of the 1060 aluminum alloy after continuous casting and rolling is shown in Figure 3. It can be seen from the figure that the grains on the upper surface of the cast rolling strips are obviously elongated and have become fibrous; on the longitudinal section of the cast rolling strips, the grains are also fibrous. In comparison, it was found that the fibrous grains of the ultrasonic cast rolling strip tested with the 0.18 wt% Al-Ti-B refiner and the ultrasonic cast rolling strip tested with the 0.12 wt% Al-Ti-B refiner were finer, with average grain sizes of approximately 39.6 μm and 34.5 μm, respectively. The average grain size of the conventional cast rolling strip tested with the 0.18 wt% Al-Ti-B refiner was about 50.2 μm, and the grain size of the ultrasonic cast rolling strip tested with the 0.09 wt% Al-Ti-B was somewhat coarse and unevenly distributed, and the average grain size was about 54.0 μm. On the cross section of the cast rolling strips, the grain size of the ultrasonic cast rolling strip tested with the 0.18 wt% Al-Ti-B refiner and the ultrasonic cast rolling strip tested with the 0.12 wt% Al-Ti-B refiner were fine and uniform, and the average grain size was about 29.5 μm and 28.6 μm, respectively. Meanwhile, the average grain size of the conventional cast rolling strip with the 0.18 wt% Al-Ti-B refiner was about 33.4 μm, and that of the ultrasonic cast rolling strip with the 0.09 wt% Al-Ti-B refiner was coarser and unevenly distributed, with an average grain size of about 35.1 μm.

It can be seen from the above comparison that under the same 0.18 wt% Al-Ti-B refiner addition, the average grain size of the ultrasonic cast rolling strip was significantly smaller than that of the conventional cast rolling strip, indicating that the ultrasonic bending vibration could effectively refine the grain microstructure of the cast rolling strips. When the Al-Ti-B refiner addition was reduced to 0.12 wt%, the average grain size of the ultrasonic cast rolling strip was still smaller than that of the conventional cast rolling strip, but when the Al-Ti-B refiner addition was reduced to 0.09 wt%, the grain sizes of the ultrasonic cast rolling strip also became coarse and uneven, and the average grain size exceeded that of the conventional cast rolling strip with 0.18 wt% Al-Ti-B refiner.

**Figure 4:**
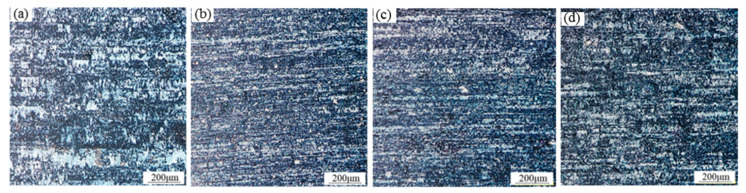
Grain structure after the subsequent cold rolling of the 1060 aluminum alloy cast rolling strip: (**a**) the top surface of the strip with added 0.18 wt% Al-Ti-B refiner and applied conventional treatment; (**b**) the top surface of the strip with added 0.18 wt% Al-Ti-B refiner and applied ultrasonic treatment; (**c**) the top surface of the strip with added 0.12 wt% Al-Ti-B refiner and applied ultrasonic treatment; (**d**) the top surface of the strip with added 0.09 wt% Al-Ti-B refiner and applied ultrasonic treatment.

The microstructure of the 1060 aluminum alloy cast rolling strip after the subsequent cold rolling is shown in Figure 4. Compared with the conventional cold rolling strip, the ultrasonic cast rolling strip with 0.18 wt% Al-Ti-B refiner or 0.12 wt% Al-Ti-B refiner had an obvious fiber structure that was finer and more uniform, without obvious blocks; the 0.18 wt% Al-Ti-B refiner-added ultrasonic cast rolling strip had the finest fiber structure after cold rolling. However, the 0.09 wt% Al-Ti-B refiner-added ultrasonic cast rolling strip had the tendency to coarsen the fiber grains after cold rolling.

### 3.2. Effect of the Ultrasonic Bending Vibration on the Precipitated Phases of the 1060 Aluminum Alloy Cast Rolling Strip and Subsequent Cold Rolling Strip

**Figure 5:**
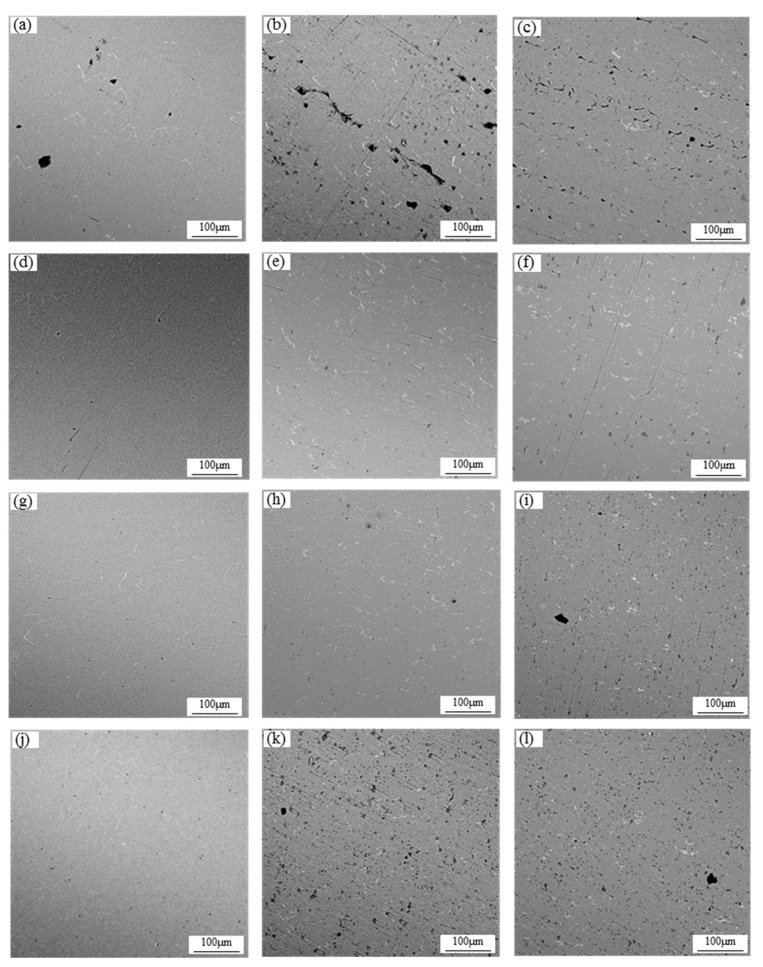
Precipitated phases of the 1060 aluminum alloy cast rolling strips: (**a**–**c**) the top surface, longitudinal section, cross section of the strip with added 0.18 wt% Al-Ti-B refiner and applied conventional treatment; (**d**–**f**) the top surface, longitudinal section, cross section of the strip with added 0.18 wt% Al-Ti-B refiner and applied ultrasonic treatment; (**g**–**i**) the top surface, longitudinal section, cross section of the strip with added 0.12 wt% Al-Ti-B refiner and applied ultrasonic treatment; (**j**–**l**) the top surface, longitudinal section, cross section of the strip with added 0.09 wt% Al-Ti-B refiner and applied ultrasonic treatment.

The precipitated phases on the surface, longitudinal section, and cross section of the 1060 aluminum alloy cast rolling strips under different treatments are shown in Figure 5. It can be seen that on the upper surface, longitudinal section, and cross section, the precipitated phase of the conventional cast rolling strip with 0.18 wt% Al-Ti-B refiner is coarse, unevenly distributed, and enriched, and the slag inclusion defects are very obvious; meanwhile, the precipitated phases in the ultrasonic cast rolling strip with 0.18 wt% Al-Ti-B refiner or 0.12 wt% Al-Ti-B refiner are more dispersed and uniformly distributed, and there are no large slag inclusion defects. However, when the addition amount of the Al-Ti-B refiner is reduced to 0.09 wt%, the precipitated phases of the ultrasonic cast rolling strip tend to be coarsened and the slag inclusion defects increase. This shows that the ultrasonic bending vibration can make the precipitated phases of the 1060 cast rolling strip disperse and uniformly distributed to a certain extent, and can significantly reduce the slag inclusion defects in the cast rolling strip.

**Figure 6:**
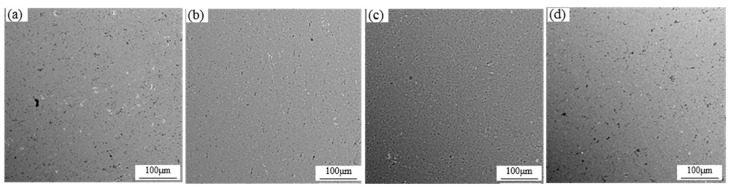
The precipitated phases of the subsequent cold rolling of the 1060 aluminum alloy cast rolling strip: (**a**) the top surface of the strip with added 0.18 wt% Al-Ti-B refiner and applied conventional treatment; (**b**) the top surface of the strip with added 0.18 wt% Al-Ti-B refiner and applied ultrasonic treatment; (**c**) the top surface of the strip with added 0.12 wt% Al-Ti-B refiner and applied ultrasonic treatment; (**d**) the top surface of the strip with added 0.09 wt% Al-Ti-B refiner and applied ultrasonic treatment.

The precipitated phases on the surfaces of the 1060 aluminum alloy cast rolling strips after subsequent cold rolling under different treatments are shown in Figure 6. Compared with the subsequent cold rolling of the strip with conventional treatment, the ultrasonic cast rolling strip after cold rolling has a fine dispersed phase, a regular shape, and a relatively uniform distribution. This indicates that the solute elements in the material have uniformly diffused into the aluminum matrix, which can reduce the degree of component segregation. However, the size, shape, and distribution of the precipitated phases are not uniform, and there is an obvious solute enrichment area in the conventional cast rolling strip with the 0.18 wt% Al-Ti-B refiner addition after cold rolling. In addition, compared with the three types of ultrasonic cast rolling strips with the addition of the Al-Ti-B refiner, the ultrasonic cast rolling strip with added 0.18 wt% Al-Ti-B refiner or 0.12 wt% Al-Ti-B refiner has fewer slag inclusion defects after the cold rolling.

### 3.3. The Effect of the Ultrasonic Bending Vibration on the Mechanical Properties of the 1060 Aluminum Alloy Cast Rolling Strips and Subsequent Cold Rolling Strips

The test results of the mechanical properties of the 1060 aluminum alloy cast rolling strips under four treatment conditions are shown in Table 2. It can be seen that in terms of tensile strength, adding the 0.18 wt% Al-Ti-B to the ultrasonic cast rolling strip improves the tensile property by 2%, while the other three have no significant difference. In terms of yield strength, compared with the addition of the 0.18 wt% Al-Ti-B refiner to the conventional cast rolling strip, the addition of the 0.18 wt% Al-Ti-B refiner, 0.12 wt% Al-Ti-B refiner, and 0.09 wt% Al-Ti-B refiner to the ultrasonic cast rolling strip caused a decrease of 0.98%, 0.98%, and 1.83%, respectively. In terms of elongation, compared to adding the 0.18 wt% Al-Ti-B refiner to the conventional cast rolling strip, adding the 0.18 wt% Al-Ti-B refiner, 0.12 wt% Al-Ti-B refiner, and 0.09 wt% Al-Ti-B refiner to the ultrasonic cast rolling strip caused an improvement of 6.42%, 2.73%, and 2.71%, respectively. Similarly, percentage reduction of area also increased by 2%, 2.52% and 1.57%, respectively. In summary, under the action of the ultrasonic bending vibration, the mechanical properties of the cast rolling strips have been improved to a certain extent. When the addition amount of the Al-Ti-B refiner was 0.18 wt%, the mechanical properties of the cast rolling strip had the greatest improvement.

The mechanical properties of the 1060 aluminum alloy cast rolling strip after cold rolling under four treatment conditions are shown in Table 3. It can be seen that in terms of tensile strength, compared with the conventional cast rolling strip with added 0.18 wt% Al-Ti-B refiner after cold rolling, the addition of the 0.18 wt% Al-Ti-B refiner, 0.12 wt% Al-Ti-B refiner, and 0.09 wt% Al-Ti-B refiner to the ultrasonic cast rolling strip after cold rolling all caused an improvement of 6.36%, 5.76%, and 1.68%, respectively. In terms of elongation, compared with the addition of the 0.18 wt% Al-Ti-B refiner to the conventional cast rolling strip after cold rolling, the addition of 0.18 wt% Al-Ti-B refiner to the ultrasonic cast rolling strip after cold rolling caused an increase of 4.11%, but the addition of the 0.12 wt% Al-Ti-B refiner or 0.09 wt% Al-Ti-B refiner to the ultrasonic cast rolling strip after cold rolling caused a reduction of 8.9% and 4.79%, respectively. This shows that the effect of the ultrasonic bending vibration on the mechanical properties of the cast rolling strip has been extended after the cold rolling, which is beneficial to the further processing of the cold rolling strip.

### 3.4. Discussion

According to the experimental research in this paper, it can be concluded that the ultrasonic bending vibration improves the refinement efficiency of the Al-Ti-B refiner. Compared with the conventional casting and rolling strip with the 0.18 wt% Al-Ti-B refiner, the grain size of the ultrasonic casting and rolling strip with the 0.18 wt% Al-Ti-B refiner or 0.12 wt% Al-Ti-B refiner are smaller, the precipitated phases are dispersed, the distribution is uniform, and the mechanical properties are also improved. Moreover, after the cold rolling of the casting and rolling strip, the effect of the ultrasonic bending vibration on the improvement of the microstructure and properties of the aluminum alloy strip is inherited.

Al-Ti-B refiner plays an important role in the grain refinement of the aluminum alloy, and the refinement effect of the refiner is largely determined by the size and shape of the TiB_2_ particles and TiAl_3_ phase. The fine and uniformly distributed TiAl_3_ phase and the dispersed and isolated TiB_2_ particles are all helpful in improving the refining effect of the refiner [29,30]. TiB_2_ particles inoculated into the melt through the Al-Ti-B master alloys are able to enhance the heterogeneous nucleation of α-Al grains [31,32,33]. However, TiB_2_ particles are small in size and easy to aggregate into clusters, which are difficult to separate when added into the melt metal. Due to their high density and being easy to deposit, TiB_2_ particles basically lose the role of the nucleation core, reducing the refining efficiency and becoming inclusions, thus reducing the quality of the aluminum products [34,35].

The ultrasonic bending vibration induced by the L-shaped ultrasonic rod into the aluminum melt in the launder will produce a significant acoustic flow effect, which will produce an obvious stirring and scouring effect in the aluminum melt. This causes the TiB_2_ particles to be evenly distributed in the aluminum melt, reduces the aggregation and precipitation of the TiB_2_, and makes the effect of the grain refinement more obvious. At the same time, the stirring effect of the ultrasonic bending vibration can promote the diffusion of the alloy elements in the aluminum melt and solid solution in the aluminum matrix, reduce the micro segregation, and inhibit the formation of coarse compounds, thus strengthening the matrix.

However, when the amount of Al-Ti-B refiner is reduced from 0.18 wt% to 0.09 wt%, even if it has the effect of the ultrasonic bending vibration the microstructure and properties of the 1060 aluminum alloy cast rolling strip and its subsequent cold rolling strip will deteriorate. This shows that the ultrasonic energy applied in the continuous casting and rolling process of the 1060 aluminum alloy in this paper cannot completely replace the role of the Al-Ti-B refiner. However, it can also be found from the text that when the amount of Al-Ti-B refiner is reduced from the conventional 0.18 wt% to 0.12 wt%, the microstructure and properties of the 1060 aluminum alloy cast rolling strip and its subsequent cold rolling strip are better than those of the conventional. The ultrasonic energy applied in this paper can reduce by the 1/3 amount of Al-Ti-B refiner used during continuous casting and rolling of the 1060 aluminum alloy, which is conducive to reducing the production costs.

## 4. Conclusions

(1) When the ultrasonic bending vibration is applied to the 1060 aluminum alloy during continuous casting and rolling under the condition of the same amount of Al-Ti-B refiner, the grains of the cast rolling strip are refined, the precipitated phases are dispersed and evenly distributed, the defects are obviously reduced, and the mechanical properties of the cast rolling strip are improved. The microstructure and properties of the ultrasonic cast rolling strip with 0.18 wt% Al-Ti-B refiner or 0.12 wt% Al-Ti-B refiner are better than those of the conventional cast rolling strip, but the microstructure and properties of the ultrasonic cast rolling strip with the 0.09 wt% Al-Ti-B refiner are worse than those of the conventional cast rolling strip.
(2) The microstructure and properties of the 1060 aluminum alloy ultrasonic cast rolling strips after cold rolling with 0.18 wt% Al-Ti-B refiner or 0.12 wt% Al-Ti-B refiner are better than those of the conventional cast rolling strip after cold rolling, but the microstructure and properties of the ultrasonic cast rolling strip with added 0.09 wt% Al-Ti-B refiner are worse than those of conventional cast rolling strip. This indicates that the effect of the ultrasonic bending vibration on the improvement of the microstructure and properties of the aluminum alloy strips is inherited during the cold rolling process, which is conducive to further processing.
(3) Applying the ultrasonic bending vibration during the continuous casting and rolling of the 1060 aluminum alloy can reduce the addition of Al-Ti-B refiner by 1/3. The L-shaped ultrasonic rod is resistant to high temperatures, and the transducer is not affected by the direct high temperature radiation of the aluminum melt. In addition, the nano ceramic radiator will not be eroded by long-term contact with the aluminum melt, which can realize continuous industrial production and reduce the production costs of industrial production.

## Figures and Tables

**Table 1:** Chemical compositions of the 1060 aluminium alloy (wt%).

Si	Cu	Mg	Zn	Mn	Ti	V	Fe	Al
≤0.25	≤0.05	≤0.05	≤0.05	≤0.05	≤0.03	≤0.05	0–0.4	Bal.

**Table 2:** Mechanical properties of the 1060 aluminum alloy cast rolling strips with different treatments.

Samples	Direction	Tensile Strength/MPa	Yield Strength/MPa	Elongation/%	Reduction in Area/%
0.18 wt% Al-Ti-B conventional	RD (cast rolling direction)	91.26	64.15	49.8	85.79
	45°	84.66	62.46	46.16	88.12
	TD (transverse cast rolling direction)	90.54	65.57	49.24	85.69
0.18 wt% Al-Ti-B ultrasound	RD	93.46	64.18	51.28	87.98
	45°	86.02	65.05	46.48	91.58
	TD	92.25	61.06	56.76	85.18
0.12 wt% Al-Ti-B ultrasound	RD	91.09	62.37	49.88	90.14
	45°	84.96	62.29	45.72	90.63
	TD	90.95	65.63	53.56	85.36
0.09 wt% Al-Ti-B ultrasound	RD	91.74	62.05	49.00	88.02
	45°	84.95	62.88	47.48	90.13
	TD	90.62	63.75	52.64	85.52

**Table 3:** Mechanical properties of subsequent cold rolling of the strips of the 1060 aluminum alloy cast rolling strip with different treatments.

Samples	Direction	Tensile Strength/MPa	Yield Strength/MPa	Elongation/%
0.18 wt% Al-Ti-B conventional	RD	150.73	148.10	2.92
	45°	145.06	141.40	3.8
	TD	153.51	153.00	2.04
0.18 wt% Al-Ti-B ultrasound	RD	167.02	165.48	2.96
	45°	153.5	149.58	3.08
	TD	157.38	156.61	3.08
0.12 wt% Al-Ti-B ultrasound	RD	165.4	165.15	2.61
	45°	156.75	154.94	3.25
	TD	153.02	153.02	2.12
0.09 wt% Al-Ti-B ultrasound	RD	152.85	152.22	1.85
	45°	149.7	145.92	2.52
	TD	154.3	153.53	3.96
